# Agility performance in healthy older adults is associated with handgrip strength and force development: results from a 1-year randomized controlled trial

**DOI:** 10.1007/s41999-023-00789-8

**Published:** 2023-05-09

**Authors:** Berit K. Labott, Lars Donath

**Affiliations:** Department of Intervention Research in Exercise Training, Institute of Exercise Training and Sport Informatics, German Sport University Cologne, Am Sportpark Müngersdorf 6, 50933 Cologne, Germany

**Keywords:** Muscle strength, Training intervention, Physical performance, Community-dwelling

## Abstract

**Purpose:**

Handgrip strength is considered as important indicator for general fitness in older adults. However, it does not notably reflect adaptations from whole-body training but may reflect adaptions of multicomponent exercise training. These approaches seem to be more functional and related to relevant daily tasks. Effects of multicomponent agility training on handgrip strength are analysed.

**Methods:**

Healthy older adults (*N* = 79, 69.3 ± 4.4 years, 64% female) were randomly assigned to an intervention (IG) or control group (CG). IG took part in a twice weekly 60 min multicomponent agility training for 12 months. Adherence rate of the participants was 75 ± 10 %.

**Results:**

Neither maximum handgrip strength (F_max_) differed between groups (IG: 318 ± 97 N, CG: 302 ± 92 N) nor did it change after the intervention (IG: 315 ± 90 N, CG: 301 ± 97 N). Mixed ANOVA for F_max_ (*F*(1,49) = 0.018,* p* = 0.893) revealed no significant group × time interaction with an effect size of $\eta^2 \leq 0.001$. Similar results were observed for rate of force development (RFD) (*F*(1,49) = 0.038, *p* = 0.847) with an effect size $\eta^2 = 0.001$ . RFD did not differ between groups in pre (IG: 876 ± 585 N/s, CG: 712 ± 303 N/s) and post (IG: 890 ± 424 N/s, CG: 702 ± 368 N/s) measurements. Correlation for ACE and F_max_ (*r*(64) = − 0.367, *p* = 0.005) and for RFD (*r*(64) = − 0.487, *p* < 0.001) was found to be negative.

**Conclusion:**

A 1-year multicomponent agility training does not affect handgrip strength in healthy older adults. However, handgrip strength (F_max_ and RFD) is associated with agility, thus both handgrip strength indicators and agility might serve as local and functional vitality surrogates.

## Key summary points

**Aim:**

Does a multicomponent agility training improves handgrip strength (maximum force and rate of force development) in healthy older adults and what is the link between handgrip strength dimensions and agility in healthy older adults?

**Findings:**

Neither maximum handgrip strength nor rate of force development of handgrip strength in healthy older adults is influenced by a 1-year multicomponent agility training. However, maximum handgrip strength and rate of force development are associated with agility performance measured via the agility challenge for the elderly.

**Message:**

Handgrip strength is not influenced by multicomponent agility training but could serve as an indicator for agility performance in older adults.

## Introduction

Measuring maximum isometric handgrip strength is common in the field of gerontology as it is considered as an important vitality surrogate for general fitness, cognitive status, frailty and sarcopenia in older adults [1–4]. Handgrip strength is furthermore a strong predictor for mortality, mobility and disability [5, 6]. It is non-invasive, cost-effective, easy to administer in different populations and provides information about general body strength and nutritional status [7, 8]. By conducting isometric handgrip strength measurements with a hand dynamometer, the maximum (F_max_) exerted force is commonly analysed. Using other measurement system e.g. load cells together with software to record the time course of the exerted force the rate of force development (RFD) can be analysed [9, 10]. This can be of special interest in older adults, as during the ageing process not only muscle mass and strength decrease [11, 12] but also neurological change of muscle function occur and affects maximum voluntary strength exertion and the capacity of rapid force exertion [13].

The effect of general exercise training and physical activity on handgrip strength is merely small to moderate. A meta-analysis of our group revealed that handgrip strength does not notably reflect adaptations from whole-body resistance training in healthy older adults [14] and it is not recommended to be used for all types of exercise modes as a measurement of effectiveness of training interventions. The meta-analysis, however, states that functional multicomponent training approaches could possibly influence handgrip strength in healthy older adults [14].

Physical activities, even when not directly affecting handgrip strength, with low intensity, can lead to important health benefits and risk reductions of both non-communicable diseases and aging-associated diseases [15]. The World Health Organization [16] and the American College of Sports Medicine [17] recommend different types of training as strength, endurance, and balance training in order to induce a broad range of adaptions improving health. Multicomponent exercise training approaches combine those different elements and could be timesaving. An exercise approach including multicomponent exercises and integrating agility components seem to be more functional and related to relevant tasks of daily life as it includes e.g., stop and go, change of direction, cutting and reactive and decision-making demands [18]. The terminology employed in this discussion revolves around “agility”, as defined by Donath, Dieen, and Faude [18] as a specific training approach with accelerations, decelerations, stop-and-go patterns, changes of direction (cutting manoeuvres), and eccentric loads, combined with demanding spatial orientation tasks. While Sheppard and Young [19] have previously defined agility as “a rapid whole-body movement with change of velocity or direction in response to an external stimulus”, the gerontological literature commonly employs concepts such as intrinsic capacity and physical function. These concepts encompass both physical and cognitive abilities within the individual [20].

The results from the previous meta-analysis of our working group [14] in addition to the idea of a novel multicomponent agility approach to train functional tasks for daily living [18] lead to the hypothesis that multicomponent agility training could improve also handgrip strength in the healthy population.

Thus, the present RCT aims at analysing the effects of a multicomponent agility training approach on handgrip strength in healthy older adults. Furthermore, it aims analysing the correlation between the measurements of handgrip strength and agility operationalized by the agility challenge for the elderly (ACE). The hypothesis is that the multicomponent agility training improves maximum force and rate of force development of handgrip strength in healthy older adults. In addition, it is hypothesised that a higher value of maximum force and rate of force development correlates with a faster time measured for completing ACE.

## Method

The present study is a two-armed randomized controlled intervention trial over 1 year. All participants were informed about the procedures and provided informed written consent. The study is in accordance with the Declaration of Helsinki, and received ethical approval of the German Sport University (no. 31/2018). Anthropometric data, maximum strength and rate of force development of handgrip strength and agility via the agility challenge for the elderly (ACE) were assessed before and after the intervention period. All testing session were conducted according to established standard operational procedures at the laboratory and were performed at the similar time of day for each participant.

### Participants

Community-dwelling, healthy older adults aged 60 years or older were recruited through newspaper advertisement in Cologne, Germany. Inclusion criteria were retirement and independent living and they should not be engaged in two or more structured training sessions per week within 3 months before the start of the study. Furthermore, their expected time for holiday and time of travel should not exceed 2 months in total over the entire year.

Older adults were excluded from the study when they were heavy smokers (more than 15 “pack years”), their body-mass index (BMI) was above 35 kg/m^2^ and when their Mini-Mental Status Examination (MMSE) score was below 26 points. Furthermore, they were excluded when cardiovascular disease or depression was present without medical consulting. Other criteria for exclusion were chronic systemic inflammation or severe lung disease, insulin-dependent diabetes, symptomatic cancer or a cancer therapy, orthopaedic diseases except those free of symptoms, and more than mild, age-related osteoporotic changes. The a priori power-analysis to estimate the sample size [21] was conducted using G*Power. Assuming moderate effects [14] (f = 0.2) for the training-induced changes in the outcomes (statistical power 90% and two-sided significance level α = 0.05), 68 participants (34 per group) are needed for statistical analysis. Including a drop-out rate of about 25%, a total of 85 participants were initially recruited.

All participants had to accept the randomized assignment. Participants were distributed using minimization method [22]. They were randomly assigned to either the intervention (IG, *n* = 39) or control group (CG, *n* = 40) by using maximum oxygen uptake (VO_2_ max) and physical activity levels as strata. The characteristics of the participants are shown in Table 1.

**Table 1:** Characteristics of the participants

Characteristic	IG (*n* = 39)	CG (*n* = 40)
Gender, female/male (n)	25/14	25/15
Age [years], M ± SD	69.4 ± 4.3	69.2 ± 4.5
Weight [kg], M ± SD	77.4 ± 15.3	77.5 ± 15.1
Height [m], M ± SD	1.69 ± 0.1	1.68 ± 0.1
BMI [kg/m^2^], M ± SD	27.1 ± 4.1	27.2 ± 4.0
MMSE [score], M ± SD	28.5 ± 1.1	28.4 ± 1.1
VO_2_max [ml/min/kg], M ± SD	24.7 ± 5.1	25.3 ± 6.2
Physical activity [min/week], M ± SD	494 ± 303	578 ± 430
Intake of medication [number of participants (*n*)]	10	12
Intake of ≥ 2 medicines [number of participants (*n*)]	2	5

*M* mean, *SD* standard deviation, *BMI* body mass index, *MMSE* mini mental state examination

### Intervention

IG took part in multicomponent agility training (strength, coordination, start-stop movements and change of directions, dual task and decision-making tasks) twice a week on non-consecutive days for 1 year. The total training volume was around 90 sessions and adherence of the participants ranged between 56 and 91% (mean 75%, standard deviation 10%). Participants were divided into three training groups of 13 participants to be able to individually assess everyone. Two trained study assistants (male and female) with experience in exercise training for older adults supervised all training sessions. The training was held at gyms at the German Sport University and were equipped with regular basic equipment as mats, balls, cones, ropes, hoops, etc. Every session lasted 60 min, where 10 min were agility specific warming-up, 45 min of agility training and 5 min of cool-down. The detailed description of the training program can be found elsewhere [21]. The complex, multicomponent agility training includes primarily not only exercises for the lower limbs but also exercises for the upper body and the core are also implemented, furthermore dual task exercises are included. Exercises for the upper body are conducted with an elastic band and balls in order to address specifically the handgrip. Adherence for each training session was noted. CG continued participating in their normal behavioural sportive and daily activities.

### Measurements

#### Handgrip strength (HGS)

Handgrip strength (F_max_ and RFD) was assessed pre and post with a hand dynamometer (Digimax, Hamm, Germany) in a stable standing position (left leg slightly forward) exerting force with the right hand. The left hand was placed at the left hip. The handle of the dynamometer was individually adjusted based on the length of the palm of the participant and the height of the device was adjusted to the participant’s body height. The device was fixed on an adjustable table. The participant’s elbow is in that position rectangularly and the forearm parallelly positioned to the ground. Participants were instructed to first get their grip, then on command preload with some (ca. 20%) force and on the following command of the test assessor they should exert as fast and as much force as they could without changing their position (e.g. bending the trunk forward). During the generation of force the test assessor motivated the participant verbally. Three trials were performed with 60 seconds (s) of rest between the attempts. The measurement of handgrip strength in older adults is considered reliable [23].

Data were computed using the software IsoTest (version 2.0, meachTronic, Hamm, Germany). Measurements were conducted with a 100 Hz sample rate. Maximum handgrip strength measured in Newton is the highest force value participants could reach. Out of the three trials, the mean of the two best trials was used for further analysis. Rate of force development measured in Newton per seconds was analysed as the maximum slope in the force–time curve averaged over 150 ms (centered moving average). Again, the mean of the two best trials out of the three were used for further analysis.

#### Agility challenge for the elderly (ACE)

As a functional and integrative test for neuromuscular and cardiocirculatory capacity, the ACE course was assessed. The detailed description of the course and its three segments (i) start-and-stop movements, (ii) change of direction and (iii) spatial orientation can be found elsewhere [21, 24]. It is a reliable and valid testing instrument, ICC of the between-day comparison is stated as 0.93 (95% confidence interval 0.88–0.96) and ICC of within-day comparison is 0.94 (95% confidence interval 0.91–0.97) [24]. All participants did fours trails (the first one as familiarisation) with a rest in between while they were not allowed to see other participants performing. The time to complete the course was measured with photoelectric time gates (DLS/F03, Sportronic, Leutenbach-Nellmersbach, Germany) and the best out of three trials was included into further analysis.

### Statistical analysis

All data were checked for normal distribution using the Shapiro–Wilk-test. A mixed ANOVA (2 (group: IG vs. CG) × 2 (time: pre vs. post) was computed to analyse the time × group interaction effect of maximum handgrip strength and rate of force development. Post-hoc testing was done in case of a statistical significant interactions. To estimate the corresponding main or interaction effect sizes, partial eta squares ($\eta_{p}^{2}$) were additionally calculated. Thereby, $\eta_{p}^{2}$ ≥ 0.01 indicates a small effect, $\eta_{p}^{2}$ ≥ 0.059 a medium effect, and $\eta_{p}^{2}$ ≥ 0.138 a large effect [25]. All participants, that completed measurements regardless of their adherence rate, were included in the statistical analysis. A paired *t*-test was use for analysis of ACE results. Spearman correlation analysis was used for calculating the correlation between maximum handgrip strength and time to complete the ACE and between rate of force development of handgrip strength and time to complete the ACE. Cohen’s d effect sizes were provided for pairwise comparison (trivial: *d* < 0.2, small: 0.2 < *d* < 0.5, moderate: 0.5 < *d* < 0.8, large *d* > 0.8) [26]. RStudio (version 2022.02.0 + 443) was employed for statistical analysis.

## Results

### Handgrip strength

#### Maximum handgrip strength

Repeated measurements ANOVA (IG vs. CG) × (pre vs. post) for maximum handgrip strength (see Table 2) revealed no significant group × time interaction effect (*F*(1,49) = 0.018, *p* = 0.89) and furthermore indicating no effect with an effect size η^2^ ≤ 0.001.

**Table 2:** Handgrip strength parameters

	IG (*n* = 24)	CG (*n* = 27)
F_max_ [N], M ± SD		
Pre	318 ± 97	302 ± 92
Post	315 ± 90	301 ± 97
RFD [N/s], M ± SD		
Pre	876 ± 585	712 ± 303
Post	890 ± 424	702 ± 368

*M*  mean, *SD* standard deviation, *N* Newton, *s* seconds, *Fmax* maximum isometric handgrip strength, *RFD* rate of force development of handgrip strength

#### Rate of force development of handgrip strength

Repeated measurements ANOVA for handgrip strength RFD (see Table 2) revealed no significant group × time interaction effect (*F*(1,49) = 0.038, *p* = 0.847) with an effect size η^2^ = 0.001 indicating no effect.

#### ACE course

Comparing pre (M = 50.7; SD = 6.8) and post (M = 46.3; SD = 5.0) measurement, participants were significantly faster in completing the course (*t*(19) = 4.638; *p* < 0.001; *d* = 1.04) after the intervention.

#### Correlation analysis

**Fig. 1:**
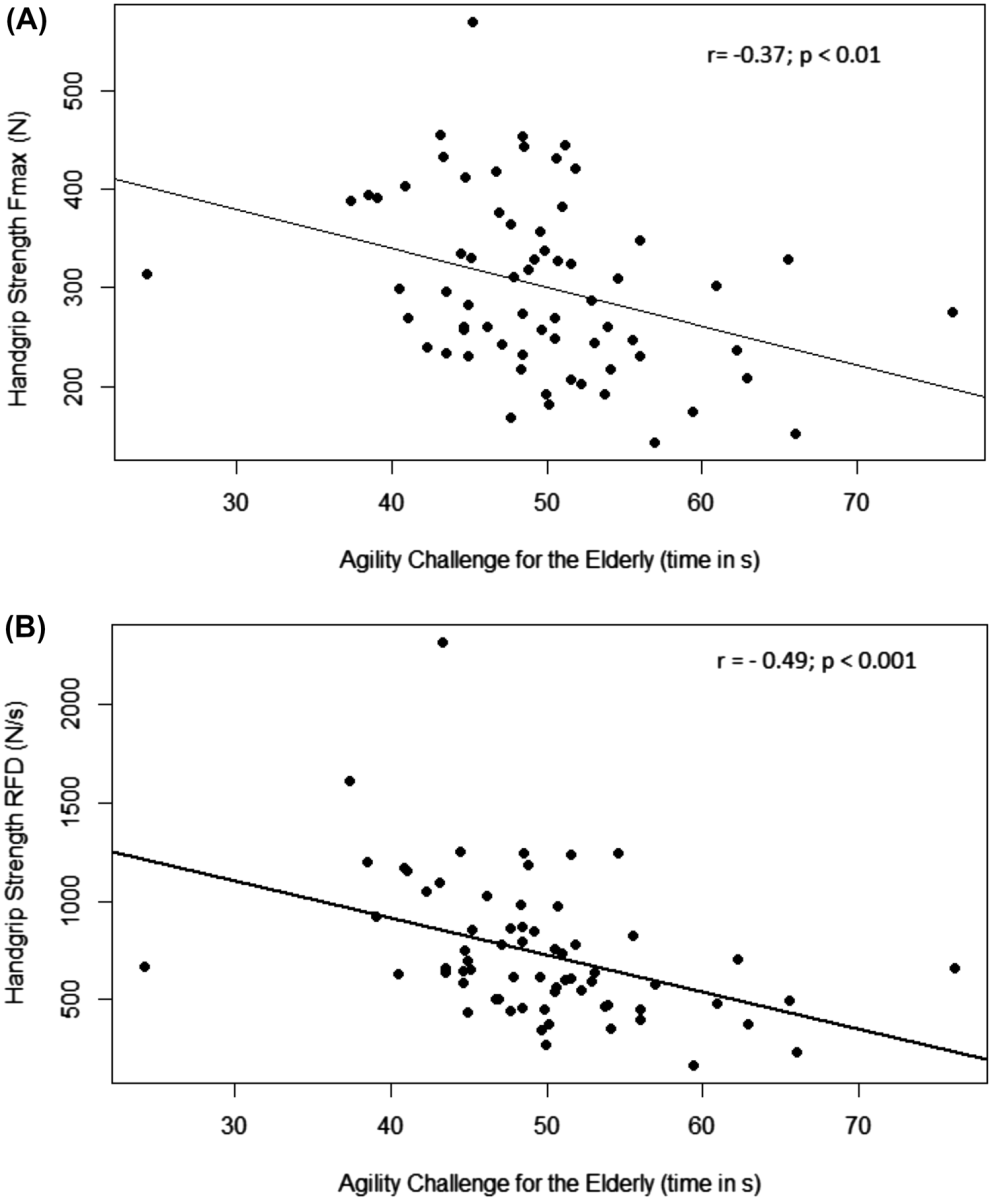
Spearman correlation for time to complete ACE challenge (in seconds) and **a** maximum handgrip strength (in N) **b** rate of force development of handgrip strength (in N/s)

Spearman correlation for ACE and maximum handgrip strength (r(64) =− 0.367, *p* = 0.005) and for RFD (r(64) =− 0.487, *p* < 0.001) was found to be significantly negative. A longer time for completing the ACE course is moderately correlated with weaker handgrip strength and a lower rate of force development (Fig. 1).

## Discussion

The present study primarily aimed at analysing effects of the multicomponent agility training on handgrip strength indicators) in healthy older adults. In addition, the effects of the multicomponent agility training on agility measured via the time to complete the ACE course was examined. Ultimately, the correlation between the measures of handgrip strength and agility (ACE) was assessed. Multicomponent agility training over 1 year does not change maximum handgrip strength or its rate of force development in healthy older adults. However, the multicomponent agility training does notably improve time to complete ACE. Correlation analysis reveals a moderate association between the measurements of handgrip strength and the time to complete ACE.

Maximum handgrip strength is not sensitive by reflecting improvements following a multicomponent agility training even when exercises for the upper body are in included. Comparisons of these results with other studies that include handgrip strength have to be conducted carefully, as there is often no standardized approach applied [27]. In line with our results are those of a multicomponent training protocol including aerobic, flexibility, muscular strength, and balance exercises for 12 weeks [28]. The participants trained 50 min in each session and the strength exercise part included three times per week exercises for the lower and upper limbs using halters, weights or elastic band, sit to stand movements from a chair, step up and down, and lunges [28]. No significant effects for handgrip strength were observed. The participants were 60 years and older and had practiced at least 1 year of regular supervised physical exercise before taking part in the study, which could be one reason for the absence of improvements in physical performance measures [28]. Another study that compared time-matched and concurrent exercise training found that handgrip strength was unchanged after 12 weeks (three times a week) of training [29]. Handgrip strength improved mainly in the concurrent aerobic and resistance exercise group compared to only aerobic and only resistance training. Participants were aged 65 years or older and performed leg press, seated rowing, chest press, lat pulldown, leg extension and triceps dips as strength exercises with an intensity of 60% of the one repetition maximum (1 RM) [29]. The aerobic exercises were conducted on a cross trainer and cycle ergometer. Timmons and colleagues stated that the results could be an indicator for the specificity of adaptation of the upper body strength and handgrip strength as it has a minor contribution during aerobic exercise but is affected by resistance training and the combined training. They reinforce the prescription for upper limb resistance exercise training in older adults for healthy ageing as it displays e.g. the risk for mortality [29]. Tieland and colleagues [30] clearly stated in their research when evaluating effects of a whole-body resistance exercise program in frail older adults that handgrip strength measurement is not a valid measurement to evaluate changes in muscle strength and also physical performance. The training consisted of exercises on machines: leg press, leg extension, chest press, lat pulldown, pec-dec, and vertical row machines starting with an intensity of 50% of the 1 RM up to 75% of 1 RM. Improvements in handgrip strength are mainly due to learning effects [30]. The status of frailty thereby did not influence the outcome of handgrip strength after the whole-body resistance training [30]. A large project (SPRINTT) with over 1000 frail older adults (mean age 78.9 years of age) delivered two multicomponent training sessions per week with additional training at home using technological support for 36 months [31]. The training consisted of moderate intense aerobic, strength, flexibility, and balance exercises for a total amount of 150 min/week. In addition, the IG received nutritional counselling. Results indicated a reduction of the risk of immobility of participants during the 36 months of follow-up period [31]. The authors reported furthermore, that women in the IG group lost less muscle strength than women in the CG. However, participants with a higher mobility score (measured via short physical performance battery) were not affected in their risk of developing a mobility disability and physical performance was slightly affected. Handgrip strength was also assessed and no significant between group differences were reported. The results are in line with the previous results and with the ones of the present study. The multicomponent exercise intervention does not influence maximum handgrip strength. A further multicomponent training of walking, balance and strength exercises over 36 month did not increase handgrip strength of sedentary older adults, the reasoning of the authors is the focus on the lower extremities, as they showed improvements in the short physical performance battery (SPPB) and 400 m walking test [32]. Even when the time period of this study is longer, similarities to the present study can be seen, as the focus was on the lower extremities. The 6-month multicomponent training intervention of Gudlaugsson et al. [33], however, showed an improvement of handgrip strength, participants walked daily (endurance training) and completed 2 weekly resistance training sessions that included 12 exercises for all major muscle groups. The first half of the strength training was strength-endurance and the last 3-month strength-power training. Exercises for lower and upper body were included, whereas exercises for the upper body consisted of bench press, chest cross, shoulder press, pull downs, biceps curls, triceps extensions [33]. A 12-week suspension exercise training also led to improvements of handgrip strength in older adults [34]; and a functional training with resistance elastic-bands including endurance, strength, balance, gross motor, and flexibility training over 20 sessions improved handgrip strength in older adults [35]. A recent meta-analysis on the effects of strength exercises in combination with other training on physical performance as handgrip strength in frail older adults concludes that a combination of strength training with other exercises improves significantly handgrip strength [36].

Assessing RFD is affected by different factors: type of instruction, method used to quantify or calculate RFD, devices used for force recording and room temperature [10]. The instruction was similar for each participant, a trained test assessor used the same wording for each measurement. Furthermore the instruction included the instruction to exert as much and as fast as possible the strength which contributes to better and valid results [37]. The RFD in handgrip strength seems also not to be influenced by non-specific exercises. Exercises for grip strength and upper extremities were present in the study (e.g., push-ups, throwing a heavy ball against the wall, resistance band rowing, squeezing tennis balls) but were not conducted with focus on a rapid conduction. A meta-analysis about the effects of resistance training on muscle strength and rate of force development in healthy older adult concludes that explosive strength training and heavy strength training are both effective methods for improving muscle strength and RFD even after a short or medium training period [38]. It was also found that muscle strength and RFD seem to adapt differently to resistance training programs [38]. Resistance training three times a week for 8 weeks mainly for the larger leg muscle groups influences the RFD of the specific muscle groups in older adults, this training was conducted with gym equipment and RFD testing was done using isometric leg press [39]. Another study also found better results of RFD after 16 weeks of training even after a detraining period of 4 weeks [40]. They included sedentary older adults and performed three sets of six to ten repetitions on an incline squat at 70–90% of 1 RM for three times per week [40]. RFD was lower after the detraining period but still significantly higher. In sedentary adults, the effect of resistance training on body composition is different compared to trained populations [41]. Other researchers found better results in RFD measurements following a heavy resistance training for 14 weeks in young adults [42]. Our training program, however, did not focus on upper extremities but more on a rapid conduction of movements of the lower extremities and did include sedentary and active healthy older adults.

The results of the ACE course in the present study are in line with the ones from the first study to assess the ACE of Lichtenstein and colleagues [24]. The results from the validity and reliability study of the ACE reveals a mean time for men of 43.1 (± 5.7) seconds and for women of 51.9 (± 4.0) seconds [24]. The researchers found that the agility course is suitable to detect changes after exercise intervention. The analysed change in the present study of the IG is 4 seconds with both (pre and post) values laying around the mean values of Lichtenstein and colleagues [24]. The hypothesis that results will be improving as reaction to familiarization and the additional training where participants exactly trained the needed skills was confirmed. The result of the improvement in the ACE course has, however, to be taken with caution as there was no control group. Interventions that assess mobility in vulnerable older adults indicated that physical activity reduces the risk for mobility disability: for example a large multicentre study in the United States was conducted to assess whether a long-term structured physical activity program for older adults is more effective than a health education program for reducing the risk of major mobility disability [43]. Participants in the intervention group completed 150-min walking per week, strength, flexibility, and balance training. They had two centre-based trainings per week and 3–4 times per week home-based activity for an average of 2.6 years. The study sample consisted of 1635 sedentary men and women (70–89 years) with physical limitations, but were able to walk 400 m. Their performance on the 400 m walking test improved and the risk of mobility impairment was reduced in the IG.

The correlation between the measures of handgrip strength and the time to complete the course is negative, meaning a higher value of maximum handgrip strength or RFD is linked to a faster time to complete the ACE. The correlation of RFD and ACE is, however, higher than the Fmax, which is in line with previous findings that RFD could be a better indicator for mobility than maximum strength alone [44, 45]. The comparison on maximum handgrip strength and rate of force development of handgrip strength and agility performance presented in this paper is only a cross-sectional contemplating.

Agility training for improving coordination, balance and reaction time through ball games, relay races, dance movements and obstacle courses in older adults improves health-related quality in older female adults [46]. Another study involving plyometric and agility training with older adults between 44 and 70 years found significant improvements in functional task abilities and agility in the training group [47]. The researchers chose to employ body-weight exercises that required to move freely through space, contrasting to machine-based resistance exercise for more functionality of exercises that match better with activities of daily living [47]. No adverse effects were reported. A previous pilot study on a similar training as in this study confirmed the feasibility of the multicomponent agility training with healthy older adults [48].

The here described training program was well received by the participants. Adherence rates were nevertheless different among participants, there was a range of 56–91%, with a mean value of 75% and a standard deviation of 10%. Reasons for missing out a training session were not recorded. The agility training included various exercises (activities with a high impact combined with challenging other activities) that could be considered as unsafe for older adults. Adverse effects or harm during the intervention did include aching muscles after training and few participants dropped out due to a lack of time or a too high intensity. These were participants that never took regularly part in physical activity. Professional supervision for a proper and safe execution of the program is needed, especially when assessing activities with a higher risk of falling or monitoring persons with a higher fall risk or insecurities. These activities can be made safer by providing support, a rail or a soft landing surface [48].

The training intervention could be carried out mostly as intended, the last training sessions in March 2020 of two groups had to be cancelled due to the restrictions of the global COVID-19 pandemic, testing was also affected and not all participants were able to finish all post-measurements. The intervention was not tailored to each individual but participants were willing adjusting the exercises to their physical fitness, e.g. a normal and a slightly more difficult option was presented and participants could choose which option was better for them. Trainers encouraged participants to try out their abilities.

One limitation of this study is a high drop-out for the post-measurements due to the worldwide pandemic. Another limitation is that the test assessors were not blinded. The present study was conducted with healthy older adults, participants with several clinical conditions and diseases were excluded from this study even when those diseases are common in the age group. The participants were more or less trained, this could lead to no adaptions in more trained individuals and more adaptions in the less trained participants. The training program, however, can be adapted to intensity and duration when tested with other populations. A strength of the study is the training period over 1 year and the relatively low drop-out rate of participants over one year.

These findings support previous findings that exercise interventions do not influence handgrip strength in older adults. The complex multicomponent agility training interventions with its specific demands has no influence on handgrip strength. It seems that handgrip strength is not a sensitive measure for adaptions of this kind of training. Handgrip strength, however, did also not decline in the present population. Handgrip strength appears to be task specific and thus seems to be sensitive to exercise interventions that focus on the explicit movement of the hand. The results suggest further that handgrip strength is not an adequate measure for training interventions. However, the results from the correlation analysis support its relation to agility, particularly regarding RFD. Future studies should investigate if vulnerable or detrained populations can improve handgrip strength, agility and mobility after multicomponent agility training as the present population reported high levels of physical activity and fitness.

## Data Availability

All data analysed during this study are included in this article. Further enquiries can be directed to the corresponding author.
